# Supplementary material for: Molecular crowding effects on protein stability in a bacterial proteome
Source: Sci Rep. 2026 Jan 21;16:5908. doi: 10.1038/s41598-026-35990-9 (PMC12894976; doi:10.1038/s41598-026-35990-9)
Supplement: Supplementary file 2 — Supplementary Material 2 [file 41598_2026_35990_MOESM2_ESM.zip › AllTheTPPData/NvD86/Panobinostat_Vignette/Melting_Curves/meltCurve_Q0K7I6.pdf]

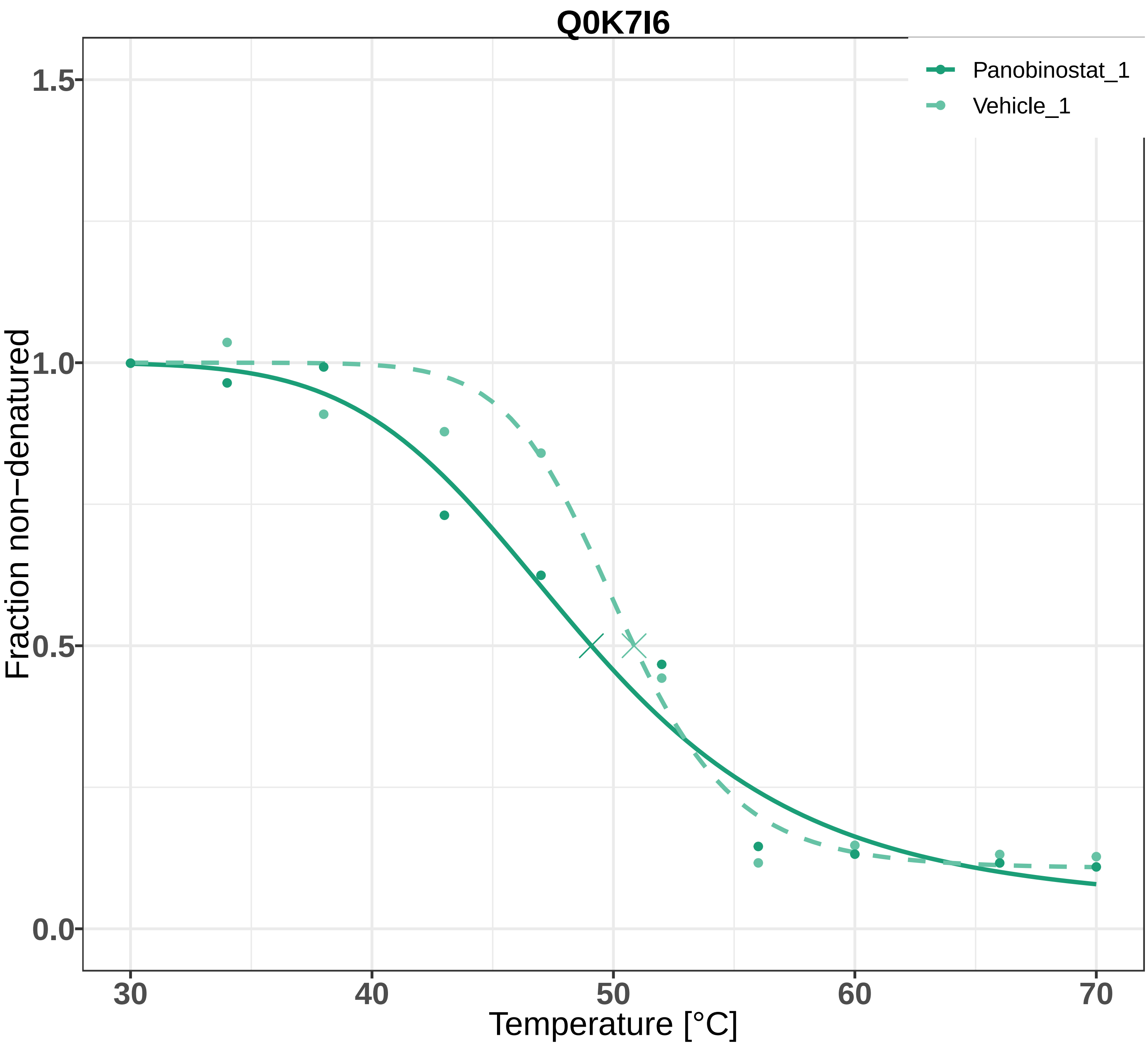


| | meltPoint | slope | plateau | R2 |
|---|---|---|---|---|
| Panobinostat_1 | 49.09 | −0.051 | 0.03 | 0.98 |
| Vehicle_1 | 50.86 | −0.095 | 0.11 | 0.98 |
